# Microalbuminuria in non-diabetic patients with unstable angina/non ST-segment elevation myocardial infarction

**DOI:** 10.1186/s13104-015-1347-x

**Published:** 2015-08-25

**Authors:** Hilal Bahjet Al-Saffar, Hussein Nassir, Anna Mitchell, Sebastian Philipp

**Affiliations:** College of Medicine, Baghdad University, Baghdad, Iraq; Iraqi Center for Heart Disease, Baghdad, Iraq; Department of Nephrology, Essen University Hospital, University of Duisburg-Essen, Essen, Germany; Department of Cardiology, Elbeklinikum Stade, Bremervörderstr. 111, 21682 Stade, Germany

**Keywords:** Microalbuminuria, Acute coronary syndrome, Coronary artery disease

## Abstract

**Background:**

Microalbuminuria (MAU) is defined as an urinary albumin excretion rate between 20–200 mg/l or 30–300 mg/day. It is a surrogate marker for endothelial dysfunction and is independently associated with atherosclerotis in diabetic and in non-diabetic patients. We assessed the prevalence of MAU in non-diabetic patients who presented with UA/NSTEMI and the relation of MAU to the severity of coronary artery disease in patients at a cardiac care center in Iraq.

**Methods:**

Seventy non-diabetic patients referred to the Iraqi Center for Heart Disease, Baghdad, between November 1st 2010 and June 1st 2011 with the diagnosis of UA/NSTEMI were included in this study. Physical examination, ECG and echocardiography were performed on all patients. TIMI (“Thrombolysis in Myocardial Infarction”) risk score was obtained. Urine samples were collected and sent for quantitative determination of MAU. All patients underwent diagnostic coronary angiography. Data are give as mean (quantitative and percent) ± SD.

**Results:**

Fifty-three men (76 %) and 17 (24 %) women (mean age 56 ± 12 years) were investigated. Overall 37 (53 %) individuals presented with arterial hypertension and 41 (59 %) with a history of smoking. 58 patients (83 %) had ischemic ECG changes (defined as ST segment depression more than 1 mm from baseline, and/or T wave inversion), 52 (74 %) had echocardiographic findings indicative of ischemia (defined as segmental wall motion abnormalities). Twenty-one (30 %) patients tested positive for MAU. There was a significant correlation of echocardiographic signs of ischemia and MAU, (n = 20 (38 %), p < 0.01). There was a clear relationship between MAU and TIMI risk score. Additionally, MAU was more common in patients with multivessel coronary artery disease (CAD) (p < 0.001). There was no statistically significant correlation between MAU and mean age, sex, smoking, and blood pressure.

**Conclusion:**

In this analysis of patients with UA/NSTEMI we found a strong correlation of microalbuminuria with echocardiographic changes and findings in coronary angiography.

## Background

Microalbuminuria (MAU) is a common phenomenon in patients with cardiovascular disease worldwide. MAU is defined as an urinary albumin excretion rate between 20–200 mg/l or 30–300 mg/day. It can also be defined as persistent excretion of albumin in the urine at rates that are above normal range but below values detected by conventional methods including ordinary dipsticks [1]. In clinical practice MAU is used to evaluate renal impairment in patients suffering from hypertension and in diabetic patients. MAU is closely associated with cardiovascular risk factors such as age, smoking, hypertension, diabetes, dyslipidemia and lack of physical activity [2–4].

In healthy individuals, the normal range for urinary albumin excretion rate is less than 30 mg/day. Urinary albumin excretion rate is known to increase with exercise, oral protein intake, urinary tract infection, and pregnancy. Albumin excretion is, on average, 25 % higher during the day than at night, with 40 % day to day variation. Albuminuria of 300 mg/day or more indicates nephropathy.

Data from several studies over the last two decades have demonstrated that MAU is not only a predictor of diabetic complications but also a powerful independent risk factor for coronary artery disease (CAD), moreover, MAU predicts development of ischemic cardiovascular events related to the development of atherosclerosis [5].

Several pathophysiological mechanisms as to how MAU may be related to the development of atherosclerotic vascular disease, have been proposed. The currently accepted mechanism primarily involves local injury to vascular smooth muscle cells and endothelium leading to cell proliferation and increase in vascular permeability [6, 7].

## Prevalence of MAU

The prevalence of MAU among non diabetic individuals with arterial hypertension ranges from 5 to 40 %. A variety of factors are responsible for this high variability: quality of blood pressure control and associated lipid abnormalities, patient selection and inclusion criteria biases such as age, race, coexisting renal disease, techniques used for detection of MAU, sampling size, and day to day variability of albumin excretion [7].

## Pathophysiology

The exact pathophysiology as to how MAU contributes to or accelerates atherosclerosis remains to be clarified. Current understanding suggests that mechanisms of vascular injury associated with MAU differ between those with or without DM but who also have hypertension [8–10]. People with MAU have an elevated transcapillary escape rate of albumin. They also have clusters of other metabolic and non metabolic risk factors associated with CVD such as arterial hypertension, dyslipidemia, and insulin resistance [11, 12].

In persons with MAU without DM, an increase in vascular permeability is caused by alterations in the extracellular matrix; this contributes to the development of endothelial dysfunction which promotes lipid influx into the vessel wall causing atherosclerotic changes [8].

In many acute and chronic illnesses, MAU is associated with increased vascular permeability as the final common pathway involving complement activation and activation of macrophages, neutrophil granulocytes, and endothelial cells via a variety of inflammatory mediators [9].

In addition to systemic vascular alterations, individuals with DM develop local injury at the level of the glomerular membrane. As a consequence of renal albumin losses vascular permeability increases and strain is put on the liver to compensate for these losses with an increased albumin synthesis [8].

However, the role of albumin in the pathogenesis of vascular disease in non-diabetic individuals could be distinct from those with diabetes: The glycation of albumin in diabetes transforms it into an antigenic molecule that initiates a variety of cellular and immune reactions, such as activation of polymorphonuclear leukocytes. Additionally, there is direct injury of glomerular membrane selectivity [13].

In summary pathophysiological processes associated with MAU are manifold: Local changes in the kidneys such as increased intraglomerular capillary pressure, increased shunting of albumin through glomerular membrane pores and loss of glomerular membrane charge; systemic changes include activation of inflammatory mediators, increased transcapillary escape rate of albumin and vascular endothelial dysfunction.

### Microalbuminuria and coronary artery disease

MAU reflects widespread vascular disease and is associated with the presence of unfavorable risk profile and target organ damage, especially in DM.

### Major risk factors for coronary artery disease in the context of MAU

#### Hypertension

Several studies have shown that the amount of MAU present in a given individual is proportional to the systolic, diastolic, and mean blood pressure as measured by either clinic or 24 h ambulatory blood pressure monitoring [11, 14]. Circadian blood pressure abnormalities, as seen in (non dippers) who are known to be at high risk for coronary artery disease, have also been described in individuals with MAU.

Men with MAU showed a higher relative risk of having an elevated systolic blood pressure compared to women with MAU. Furthermore the association of MAU and hypertension in diabetics is greater than in non diabetics [15].

#### Hyperinsulinemia

Both hyperinsulinemia and MAU have been shown to increase CVD risk in non-diabetics. This is even more true, if they coincide with other risk factors for atherosclerosis such as arterial hypertension, dyslipidemia, and obesity. Collectively, these risk factors are called syndrome X or cardiometabolic syndrome [16, 17].

#### Endothelial dysfunction

The endothelium produces components of extracellular matrix and a variety of proteins that play an important role in vascular and renal function. Impaired endothelial antithrombotic and vasodilatory properties are main factors in atherogenesis [13]. It has been proposed that defective endothelial permeability may be the origin of MAU in the general population, in those with arterial hypertension and among those with diabetes [10, 13].

Endothelial dysfunction seems to play a key role in non-diabetic glomerulosclerosis and atherosclerosis. Increased permeability of the endothelium allows atherosclerotic lipoprotein particles (oxidized LDL and others) to penetrate into the vessel wall and promote the development of atherosclerotic plaques [10, 13].

MAU is also associated with biochemical indices of endothelial dysfunction, such as an increased von-Willebrand-Factor (VWF) and increased platelet adhesiveness. Higher levels of VWF were found in individuals with MAU and a direct correlation between these two variables was described. Other biochemical indices of endothelial dysfunction also associated with MAU include elevation in plasma concentrations of angiotensin II, tissue type plasminogen activator inhibitor-1, and endothelin-1 [13]. A greater amount of MAU not only represents endothelial damage but is also associated with an adverse cardiovascular prognosis [18].

#### Dyslipidemia

Several studies have shown an increased association between MAU and abnormalities in serum lipoproteins. These lipid abnormalities include low levels of HDL as well as high levels of LDL, total triglycerides, and lipoprotein a. The most consistent association between lipoprotein abnormalities and MAU is a low level of HDL [11, 19].

### Clinical implications

The presence of MAU is of great diagnostic value since MAU represents a very sensitive manifestation of abnormal vascular permeability. Its applications as a marker of target organ damage from cardiovascular disease include risk assessments, evaluation of disease severity and prognosis [10].

From the available data, measurement of MAU is a very sensitive tool in the presence of inflammatory processes including coronary artery disease and other acute inflammatory states, such as trauma, sepsis and surgery. The amount of MAU is proportional to the severity of the condition [12]. Ischemia and reperfusion are other conditions that follow this rule [10].

MAU is also detected in the presence of an acute coronary syndrome or peripheral vascular disease, it is proportional to the severity of the infarct size or claudication [20, 21].

Early identification of MAU may influence the aggressiveness of management and ultimately the outcome of the disease.

## Patients and methods

In this study 70 patients were admitted to the coronary care unit of the Iraqi Center for Heart disease, Baghdad, with the diagnosis of UA/NSTEMI between November 1st, 2010 and June 1st, 2011. Twenty healthy persons served as a control group.

Patients with DM, STEMI, chronic stable angina, urinary tract infection, and macroalbuminuria by urinary dipstick were excluded from the study.

A detailed medical history, thorough clinical examination with particular attention to the cardiovascular system, ECG and echocardiography were performed in each patient. The study complied with the provisions of the Declaration of Helsinki regarding investigations in humans, and was approved by local ethic committees (Ministry of Health, Republic of Iraq, Medical City) and written informed consent was obtained for all patients.

TIMI risk score for UA/NSTEMI was obtained for all patients. TIMI risk score identifies seven independent risk factors for UA/NSTEMI [22]. (Given one point for each factor, scores from 0 to 7):

1. Age older than 65 years.
2. Equal or more than 3 CAD risk factors.
3. Documented CAD at catheterization (more than 50 % stenosis).
4. ST-segment deviation more than 0.5 mm from presenting ECG.
5. Equal or more than 2 anginal episodes in prior 24 h.
6. Aspirin within prior week.
7. Elevated cardiac biomarkers.

A morning urine sample was collected from each patient. Macroalbuminuria was excluded with ordinary urinary dipstick testing [23]. Urinary tract infection was included by genitourinary examination. The probes were centrifuged, the precipitant was discarded and the supernatant stored at −20 °C. MAU was tested in all samples simultaneously by immunometric enzyme immunoassay for the quantitative determination of MAU [24] by a kit from ORGENTEC diagnostika GmbH, Mainz, Germany.

In studies with urine samples from healthy individuals a range of albuminuria between 0 and 25 mg/ml has been established. Values above this range are considered pathological.

Coronary angiography was performed in all patients. Lesions >70 % stenosed were defined as significant.

Statistical Package for Social Science (SPSS 14) was used for data storage and analysis. Data are give as mean (quantitative and percent) ± SD, associations between different variables were analyzed using Chi Square test. A *p* value ≤ 0.05 was considered to be significant.

### Control group

20 age and sex matched individuals without hypertension or diabetes (10 men, 10 women) served as control. Out of these 5 (25 %) had a smoking history (4 men, 1 woman).

## Results

Seventy patients were included in this study, mean age 56 years ± 12 (range 26–89). Patient characteristics are shown in Table 1. MAU test was positive in 21 (30 %) of patients from the studied sample. Among patients with a positive MAU test (n = 21), the mean age was 55 years (Table 2). Specific differences in gender and cardiovascular risk factors as smoking and hypertension are shown in Tables 3, 4 and 5 respectively. Patients with ischemic changes in the ecg (as defined above) were more likely being MAU positive (32 versus 68 %) compared to patients without ecg changes (non-ischemic; 17 versus 83 %) (Table 6). The same we were able to find in the echo findings, even reaching here statistical significance (p < 0.001) (Table 7).

**Table 1:** Demographical characteristics of studied population

Variables	No. (%)
Age (mean ± SD)	56.51 year ± 12.85
Men	53 (76 %)
Women	17 (24 %)
Smoker	41 (59 %)
Hypertension	37 (53 %)
Ischemic ECG changes	58 (83 %)
Ischemic ECHO finding	52 (74 %)

**Table 2:** Relation between mean age and MAU

1	N	Mean(year)	SD
−ve group	49	56.48	12.57
+ve group	21	55.71	12.02

P value = 0.8

**Table 3:** Relation between sex and MAU

Factor	MAU		Total
	+ve	−ve	
Men	17 (32 %)	36 (68 %)	53 (100 %)
women	4 (24 %)	13 (76 %)	17 (100 %)

P value = 0.5

**Table 4:** Relation between smoking and MAU

Factor	MAU		Total
	+ve	−ve	
Smoker	12 (29 %)	29 (71 %)	41 (100 %)
Non-smoker	9 (31 %)	20 (69 %)	29 (100 %)

P value = 0.8

**Table 5:** Relation between hypertension and MAU

Factor	MAU		Total
	+ve	−ve	
Hypertensive	8 (22 %)	29 (78 %)	37 (100 %)
Non hypertensive	13 (39 %)	20 (61 %)	33 (100 %)

P value = 0.1

**Table 6:** Relation between ischemic ECG changes and MAU

Factor	MAU		Total
	+ve	−ve	
Ischemic changes	19 (32 %)	39 (68 %)	58 (100 %)
Non-ischemic	2 (17 %)	10 (83 %)	12 (100 %)

P value = 0.4

**Table 7:** Relation between ischemic ECHO finding and MAU

Factor	MAU		Total
	+ve	−ve	
Ischemic finding	20 (38 %)	32 (62 %)	52 (100 %)
Non-ischemic	1 (6 %)	17 (94 %)	18 (100 %)

P value = 0.0008

Patients tested MAU-positive had either intermediate or high TIMI risk scores (as shown in Table 8). Furthermore MAU positive patients had more diseased vessels. Statistic comparison between 2 groups according to MAU status considered highly significant, p < 0.001 (Table  9).

**Table 8:** Relation between MAU and TIMI risk score

TIMI score	MAU		Total
	+ve, n (%)	−ve, n (%)	
<2	(0)	16 (33 %)	16 (23 %)
3	(0)	26 (53 %)	26 (37 %)
4	11 (53 %)	6 (12 %)	17 (24 %)
5	7 (33 %)	1 (2 %)	8 (12 %)
>6	3 (14 %)	(0)	3 (4 %)
Total	21 (100 %)	49 (100 %)	70 (100 %)

**Table 9:** Relation between number of diseased coronary vessels and MAU

Factor	MAU	
	+ve	−ve
Single vessel	1 (3 %)	28 (97 %)
2 vessels	6 (26 %)	17 (74 %)
3 vessels	14 (78 %)	4 (22 %)

P value = 0.000001

In patients tested positive, the level of MAU ranged from 25.5 to 40.6 mg/ml, with cutoff point at microalbuminuric level of 29 mg/ml, above cutoff point, none of patients with 1-VD, 2 (33 %) with 2-VD, and 13 (93 %) with 3-VD, while below the cutoff point, one patient (100 %) had a 1-VD, 4 (67 %) a 2-VD, and 1 (7 %) a 3-VD respectively, p = 0.01 (Table 10).

**Table 10:** Relation between MAU level and the number of diseased coronary vessels at cutoff point = 29 µg/ml

Factor	Cutoff point = 29 µg/ml	
	Below	Above
1 VD	1 (100 %)	0
2 VD	4 (67 %)	2 (33 %)
3 VD	1 (7 %)	13 (93 %)

P value = 0.01

All individuals in the control group had a negative MAU test (1.3–9.8 mg/ml).

## Discussion

Several studies have implicated MAU as a risk factor for cardiovascular disease, particular in diabetic patients [25–27]. Cardiovascular disease leads to acute coronary syndrome as unstable angina (UA) and non-ST-segment elevation MI (NSTEMI). In this study MAU was detected in 21 (30 %) out of 70 non-diabetic patients, admitted to the Iraqi Center for Heart disease, Baghdad with a diagnosis of UA/NSTEMI. A similar result was obtained by Klausen et al., who found that MAU is a strong determinant of coronary artery disease and death independently of age, sex, hypertension, DM, renal function, and lipid profile [28].

In our cohort we found no significant correlations of MAU with age, sex, smoking, and arterial hypertension. Smoking is a recognized cardiovascular risk factor, and it may be also related to MAU. In our study, MAU was more common in smokers than in non-smokers. Similar results were obtained by Jaun-Manuel Guizer [29], while Charles et al. could not confirm this finding [30].

Although several authors have reported the association between high systolic blood pressure and MAU [33, 34], in the present investigation, we were not able to see a similiar association in our study. A similar result was obtained by Klausen, who found that MAU is a predictor of coronary artery disease, irrespective of other risk factors including hypertension [30].

The prognostic value of MAU for CVD was first established in patients with DM [31, 32]. In non-diabetic subjects, the result from several studies have indicated that MAU is a marker of cardiovascular risk, more over, several studies have demonstrated that MAU is an independent predictor of cardiovascular morbidity and mortality in non-diabetic populations [33, 34].

In our study, MAU was more common in patients with ischemic ECG changes than in those without. Additionally, there was a significant correlation with wall motion abnormalities as an echocardiographic sign of ischemia (p < 0.001). These findings add to results of Diercks et al., who described an increase in mortality in the subset of subjects with both ST-T segment depression and MAU [35].

It has been postulated that MAU indicates increased vascular endothelial permeability not restricted to renal vessels. This could promote foam cell formation and atherogenesis by increased leakage of lipoprotein particles into the vessel wall, an increased transcapillary albumin excretion rate, an increased plasma level of VWF, and an attenuated endothelium dependent response to vasodilator stimuli in subjects with MAU [36]. Therefore individuals with ST-T segment changes in addition to MAU could have an increased risk of enhanced progression of atherosclerosis and subsequent mortality.

The ability of MAU to predict adverse cardiovascular events is not restricted to high risk populations. In a low risk population for CVD, this fact was supported by Hillege et al. They demonstrated that MAU can predict CVD and non-CVD mortality in a general population [37].

Patients with high risk of adverse cardiovascular events as measured by the TIMI risk score were more likely to be MAU-positive compared to patients with low cardiovascular risk (Table 8).

A significant correlation between MAU and the severity of coronary artery disease as measured by the number of vessels with significant lesions was observed in this study. Patients with 2 or 3 vessels disease had a higher level of MAU than patients with single vessel disease, p < 0.001.

This study showed that by measuring MAU in non-diabetic patients with UA/NSTEMI, we can predict the severity of CAD and the risk of adverse outcome.

## Conclusion

In our study MAU was prevalent in patients with a diagnosis of UA/NSTEMI which is a marker of high risk CAD, regardless of other traditional risk factors for CVD.

## Abbreviations

CAD: coronary artery disease
CVD: cardiovascular disease
DM: diabetes mellitus
ECG: electrocardiogram
HDL: high density lipoprotein
LDL: low density lipoprotein
MAU: Microalbuminuria
NSTEMI: non-ST-elevation myocardial infarction
UA: unstable angina
VWF: von-Willebrand-Factor

## References

[1] Mogensen CE, Chachati A, Christensen A, Christensen CK (1985). Microalbuminuria: an early marker of renal involvement in diabetes. Urem Investig.

[2] Hillege HL, Janssen WM, Bak AA (2001). Prevend study group. Microalbuminuria is common, also in a nondiabetic, nonhypertensive population, and an independent indicator of cardiovascular risk factors and cardiovascular morbidity. J Intern Med.

[3] Hao G, Wang Z, Zhang L (2015). Prevalence of microalbuminuria among middle-aged population of China: A multiple center cardiovascular epidemological study. Angiology.

[4] Wang Y, Yuan A, Chen Y (2013). Correlation between microalbuminuria and cardiovascular events. Int J Clin Exp Med..

[5] Pedrinelli R, Penno G, dellomo G (1999). Microalbuminuria and transcapillary albumin leakage in essential hypertension. Hypertension..

[6] Taddei S, Virdis A, Mattei P (1995). Lack of correlation between microalbuminuria and endothelial function in essential hypertensive patients. J Hypertens.

[7] Bakris GC, Randall O, Rahman M, For the African American Study of Kidney Disease (AASK) Study Group (1998). Association between cardiovascular risk factors and glomerular filtration rate at baseline in the AASK trial. J Am Soc Nephrol.

[8] Schitz A (1997). Microalbuminuria, blood pressure, metabolic control, and renal involvement: longitudinal studies in white non-insulin-dependent diabetic patients. Am J Hypertens.

[9] Glosing P (1995). Microalbuminuria: a marker of systemic disease. Br J Hosp Med.

[10] Jensen JS (1995). Renal and systemic transvascular albumin leakage in severe atherosclerosis. Arterioscler Thromb Vasc Biol.

[11] Bigazzi R, Biauchi S (1995). Microalbuminuria as a marker of cardiovascular and renal disease in essential hypertension. Nephrol Dial Transpl.

[12] Panayioton BN (1994). Microalbuminuria: pathogenesis, prognosis and management. J Intern Med Res.

[13] Stehouwer CD, Lambet J, Donker AJ (1997). Endothelial dysfunction and pathogenesis of diabetic angiopathy. Cardiovasc Res.

[14] Pontremoli R (1996). Microalbuminuria in essential hypertension—its relation to cardiovascular risk factors. Nephrol Dial Transpl.

[15] Biauchi S, Bigazzi R, Baldari G (1994). Diurnal variation of blood pressure and microalbuminuria in essential hypertension. Am J Hypertens.

[16] Alzaid AA (1996). Microalbuminuria in patients with NIDDM: an overview. Diabetes Care.

[17] Kuusisto J, Mykkanen L, Pyorala K (1995). Hyperinsulinemic microalbuminuria: a new risk indicator for coronary heart disease. Circulation.

[18] Stehouwer CD, Nauta JJ, Zeldenrust GC (1992). Urinary albumin excretion, cardiovascular disease, and endothelial dysfunction in non-insulin dependent diabetes mellitus. Lancet.

[19] Groop PH, Viberti GC, Elliot TG (1994). Lipoprotein (a) in type 1 diabetic patients with renal disease. Diabet Med.

[20] Gosling P, Hughes EA, Regnolds TM (1991). Microalbuminuria is an early response following acute myocardial infarction. Eur Heart J.

[21] Hickey NC, Shearman CP, Gosling P (1990). Assessment of intermittent claudication by quantitation of exercise-induced microalbuminuria. Eur J Vasc Surg.

[22] Boersma E, Cohen M, Bernink PJ (2000). The TIMI risk score for unstable angina/non-ST elevation MI: a method for prognostic and therapeutic decision making. JAMA.

[23] Laffeyette RA, Perrone RD, Levey AS, Schrier RW, Gottschalk CW (1996). Laboratory evaluation of renal function. Disease of the kidney.

[24] Feldt-Rasmussen B, Dinesen B, Deckert M (1985). Enzyme immunoassay: an improved determination of urinary albumin in diabetic with incipient nephrology. Scand J Clin Lab Invest.

[25] Sharma S, Ghalaut VS, Dixit R (2013). Microalbuminuria and C-reactive protein as a predictor of coronary artery disease in patients of acute chest pain. J Cardiovasc Dis Res..

[26] Currie G, Delles C (2013). Proteinuria and ist relation to cardiovascular disease. Int J Nephrol Renovasc Dis.

[27] Won JC, Lee YJ, Kim JM (2013). Prevalence of factors associated with albuminuria in the Korean adult population: the 2011 Korea National Health and Nutrition Examination Survey. PLoS One.

[28] Klansen K, Barch-Johnsen K, Feldt-Rasmussen B (2004). Very low levels of micrialbuminuria are associated with increased risk of coronary of heart disease and death independently of renal function, hypertension, and diabetes. Circulation.

[29] Guizer J, Kornhanser C, Malacara J (2001). Renal function reserve in patients with recently diagnosed type 2 diabetes mellitus with and with out microalbuminuria. Nephron.

[30] Valmadrid CT, Klein R, Moss SE, Klein BE (2000). The risk of cardiovascular disease mortality associated with microalbuminuria and gross proteinuria in person with older-onset diabetes mellitus. Arch Intern med.

[31] Nelson RG, Bennet PH, Beck GJ (1996). Development and progression of renal disease in Pima Indians with NIDDM. NEJM.

[32] Friis T, Pederson LR (1997). Microalbuminuria in type 2 diabetic patients. A prospective follow up study. Ann Clin Biochem.

[33] Damsgaard EM, Froland A, Jorgensen OD (1990). Microalbuminuria as predictor of increased mortality in elderly people. BMJ.

[34] Yudkin JS, Forrest RD, Jackson CA (1988). Microalbuminuria as predictor of vascular disease in non-diabetic subjects. Islington diabetes survey. Lancet.

[35] Gilles FH, Diercks MD, Hans L. Hillege MD (2002). Microalbuminuria modifies the mortality risk associated with electrocardiographic ST-T segment changes. JACC.

[36] Pedrinelli R, Giampietro O, Carmassi F (1994). Microalbuminuria and endothelial dysfunction in essential hypertension. Lancet.

[37] Hilliege HL, Fidler V, Diercks GFH (2002). Urinary albumin excretion predicts cardiovascular and non cardiovascular mortality in general population. Circulation.

